# A Multiplex and Colorimetric Reverse Transcription Loop-Mediated Isothermal Amplification Assay for Sensitive and Rapid Detection of Novel SARS-CoV-2

**DOI:** 10.3389/fcimb.2021.653616

**Published:** 2021-06-29

**Authors:** Eduardo Juscamayta-López, Faviola Valdivia, Helen Horna, David Tarazona, Liza Linares, Nancy Rojas, Maribel Huaringa

**Affiliations:** ^1^ Laboratorio de Infecciones Respiratorias Agudas, Centro Nacional de Salud Pública, Instituto Nacional de Salud, Lima, Peru; ^2^ Laboratorio de Virus Respiratorios, Centro Nacional de Salud Pública, Instituto Nacional de Salud, Lima, Peru

**Keywords:** Multiplex RT-LAMP, SARS-CoV-2, COVID-19, rapid molecular tests, timely diagnosis, diagnostic accuracy, primary health care, resource-limited settings

## Abstract

Coronavirus disease 2019 (COVID-19) caused by severe acute respiratory syndrome coronavirus 2 (SARS-CoV-2) has become a major threat to public health. Rapid molecular testing for convenient and timely diagnosis of SARS-CoV-2 infections represents a challenge that could help to control the current pandemic and prevent future outbreaks. We aimed to develop and validate a multiplex and colorimetric reverse transcription loop-mediated isothermal amplification (RT-LAMP) assay using lyophilized LAMP reagents for sensitive and rapid detection of SARS-CoV-2. LAMP primers were designed for a set of gene targets identified by a genome-wide comparison of viruses. Primer sets that showed optimal features were combined into a multiplex RT-LAMP assay. Analytical validation included assessment of the limit of detection (LoD), intra- and inter-assay precision, and cross-reaction with other respiratory pathogens. Clinical performance compared to that of real-time reverse transcriptase-polymerase chain reaction (RT-qPCR) was assessed using 278 clinical RNA samples isolated from swabs collected from individuals tested for COVID-19. The RT-LAMP assay targeting the RNA-dependent RNA polymerase (*RdRp*), membrane (*M*), and *ORF1ab* genes achieved a comparable LoD (0.65 PFU/mL, CT=34.12) to RT-qPCR and was 10-fold more sensitive than RT-qPCR at detecting viral RNA in clinical samples. Cross-reactivity to other respiratory pathogens was not observed. The multiplex RT-LAMP assay demonstrated a strong robustness and acceptable intra- and inter-assay precision (mean coefficient of variation, 4.75% and 8.30%). Diagnostic sensitivity and specificity values were 100.0% (95% CI: 97.4–100.0%) and 98.6% (95% CI: 94.9–99.8%), respectively, showing high consistency (Cohen’s kappa, 0.986; 95% CI: 0.966–1.000; p<0.0001) compared to RT-qPCR. The novel one-step multiplex RT-LAMP assay is storable at room temperature and showed similar diagnostic accuracy to conventional RT-qPCR, while being faster (<45 min), simpler, and cheaper. The new assay could allow reliable and early diagnosis of SARS-CoV-2 infections in primary health care. It may aid large-scale testing in resource-limited settings, especially if it is integrated into a point-of-care diagnostic device.

## Introduction

Severe acute respiratory syndrome coronavirus 2 (SARS-CoV-2) is the viral etiological agent of a novel severe acute respiratory infection called coronavirus disease 2019 (COVID-19) that was declared a pandemic by the World Health Organization (WHO) on March 11, 2020 (Li et al., 2020; Wu et al., 2020). Since the outbreak that originated in December 2019 in Wuhan city, China (Adhikari et al., 2020), the new β-coronavirus rapidly spread globally, being responsible for more than 160 million confirmed cases and 3,339,002 deaths in over 200 countries, as of May 14, 2021 [World Health Organization (2020a), COVID-19 situation dashboard]. Thus, it has become a major threat to public health. A similar epidemiological situation is taking place in Peru, which has one of the highest mortality rates in the current COVID-19 pandemic, with Lima city being one of the major epicenters of SARS-CoV-2 infections in Peru, as of September 11, 2020 (Juscamayta-López et al., 2020; Ministry of Health, Peru, 2020). Accurate and timely diagnosis of SARS-CoV-2 infections is key to interrupting the transmission and spread of the virus. However, COVID-19 diagnosis is a challenge as there is no defined pattern of clinical signs and symptoms, with cases ranging from asymptomatic to mild, moderate, and severe (Wang L. et al., 2020). Also, COVID-19 clinical features have some resemblance to other previously reported coronavirus infections (SARS-CoV and MERS-CoV) and to other types of infections (such as influenza or the common cold) (Huang et al., 2020).

Currently, real-time reverse transcriptase-polymerase chain reaction (RT-qPCR) is being used to diagnose SARS-CoV-2 infections in public health and clinical laboratories worldwide because of its high specificity and sensitivity (Corman et al., 2020; WHO, 2020b). Although RT-qPCR is very useful for detecting the virus during the first 3 weeks of infection, its sensitivity varies according to the type of sample (93% using bronchoalveolar lavage, 72% using sputum, 63% using nasal swabs, and 32% using pharyngeal swabs) (Vandenberg et al., 2020). Furthermore, this method is time-consuming and requires complex equipment, specialized personnel, and high-cost consumables, which are scarce in resource-limited settings. This hinders effective and timely treatment of patients, increasing the risk of patient deterioration and the spread of the infection (Patel et al., 2020).

SARS-CoV-2 infection can also be diagnosed by detecting either the viral antigens or antibodies (Kubina and Dziedzic, 2020). The SARS-CoV-2 antigen assays are limited by the sensitivity, specificity, and production speed related to the diagnostic antibodies used in the assays (Thi et al., 2020), whereas serological tests are not useful for the early diagnosis of SARS-CoV-2 infections, and cross-reactions with other types of coronavirus are likely (Yong et al., 2020; Zhao et al., 2020).

An alternative method to RT-qPCR is loop-mediated isothermal amplification (LAMP), a rapid and highly specific molecular method that is being applied in the diagnosis of infectious diseases worldwide, including tuberculosis (Global Tuberculosis Programme, 2016) and malaria (Lopez-Jimena et al., 2018). LAMP amplifies DNA under isothermal conditions requiring only a *Bst* DNA polymerase (with displacement activity) and a set of four primers that recognize a total of six different regions on the target sequence. Loop primers can be added for accelerating the exponential amplification. The LAMP products are a mixture of stem-loop DNA with several cauliflower-like structures and multiple loops (Notomi et al., 2000).

LAMP is also low cost and can be easily used in primary health care units, so it has been considered as a potential assay for point-of-care diagnosis of infectious diseases (Kozel and Burnham-Marusich, 2017). A modified form of this methodology is reverse transcription loop-mediated isothermal amplification (RT-LAMP). Several studies have demonstrated the high efficiency of RT-LAMP at detecting the RNA of viral pathogens such as dengue (Ocker et al., 2016), chikungunya, Zika (Priye et al., 2017), and influenza (Ahn et al., 2019) viruses. RT-LAMP assays have been proposed for diagnostic detection of SARS-CoV-2 RNA (Thompson and Lei, 2020). Nevertheless, their performance is still subject to discussion due to the insufficient numbers of samples tested so far (Thi et al., 2020). In addition, most of these assays target single genetic sequences, which may reduce the sensitivity for SARS-CoV-2 RNA detection in samples with medium or low viral loads, leading to unreliable diagnostic results (Bulterys et al., 2020). Another limitation is the use of conventional liquid LAMP reagents, as they need a refrigerated environment for their storage, transport, and operation, otherwise their stability and test performance will be reduced (Nagaraj et al., 2018). There is an ongoing need to develop a new RT-LAMP assay that simultaneously detects multiplex targets of the SARS-CoV-2 genome in order to provide rapid and more reliable results, as well as to allow simpler diagnostic testing.

Here, we developed and tested a molecular assay based on one-step multiplex and colorimetric RT-LAMP using lyophilized LAMP reagents for detecting SARS-CoV-2 RNA in clinical RNA samples isolated from nasal and pharyngeal swabs collected from confirmed COVID-19 patients at the National Institute of Health - Peru (NIH-Peru). First, we sequenced the genomes of SARS-CoV-2 circulating in Peru. Using these sequences together with other SARS-CoV-2 genomic sequences in publicly available databases, we identified potential targets for sensitive and rapid detection of SARS-CoV-2. Then, we used 278 clinical RNA samples from individuals tested for COVID-19, who had a wide range of viral loads, to determine the sensitivity and specificity of our colorimetric RT-LAMP assay compared to rt-RT-PCR (gold standard).

Finally, based on the results, we present a new multiplex and portable RT-LAMP assay with high sensitivity and specificity, which allows the detection of SARS-CoV-2 visually (colorimetrically), accurately, and quickly (∼30 min) in primary care health units, with minimal laboratory resources. This will be very useful both for the timely management of individual cases and for guiding the implementation of public health measures, in order to avoid the spread of SARS-CoV-2 and reduce its negative impact on the population.

## Materials and Methods

### Study Design and Setting

This study was a retrospective study to develop and evaluate a colorimetric RT-LAMP assay based on multiplex targets for rapid and sensitive detection of SARS-CoV-2 RNA in clinical RNA samples. To develop the assay, we used RNA samples isolated from nasal and pharyngeal specimens collected from patients who had been tested for COVID-19 at NIH-Peru from March to May 2020. The study was conducted at NIH-Peru.

### Sample Collection and RNA Extraction

We obtained RNA from nasal and pharyngeal swabs from patients with suspected COVID-19 who were referred to the Laboratorio de Virus Respiratorios (LVR) of NIH-Peru for diagnostic confirmation by RT-qPCR (gold standard). Viral RNA from 100 μl of clinical sample had previously been extracted using the GenElute Total RNA Purification kit (MilliporeSigma, St. Louis, Missouri, USA) following the manufacturer’s protocols, eluted in 50 μl of elution buffer, and examined by RT-qPCR. The remaining RNA samples were stored at -80°C. We randomly selected RNA of clinical samples that were positive (n=139) and negative (n=139) for SARS-CoV-2 RNA according to RT-qPCR (gold standard) from March to May 2020 using the LVR’s database. The selected RNA samples were stored at -80°C until further processing.

### Whole-Genome Sequencing (WGS) of SARS-CoV-2

WGS of SARS-CoV-2 isolates (n=48) circulating in Peru was performed using an Illumina MiSeq System at NIH-Peru using a CleanPlex^®^ SARS-CoV-2 Panel (Paragon Genomics, Hayward, California, USA). These RNA samples were obtained from nasal and pharyngeal swabs collected from individuals who tested positive for SARS-CoV-2 RNA according to RT-qPCR with a CT value <25 at NIH-Peru. Trimming of low-quality reads, genome assembly, and consensus sequence construction were performed according to methods recently described by Juscamayta-López et al. (2020).

### Identification of Highly Conserved Targets in SARS-CoV-2 Genomes

Available SARS-CoV-2 genomes from around the world were downloaded from the GISAID database (https://www.gisaid.org), as of May 26, 2020. Searches were limited to the genomes with >29,000 base pairs and high coverage (<1% Ns and <0.05% single amino acid mutations not listed in other databases). In addition, sequences with insertions and deletions not verified by the sequencing laboratory were discarded. We obtained 23,413 aligned SARS-CoV-2 genomes. All sequences, including those from Peru, were analyzed using BioEdit v7.2 to identify highly conserved regions to be used as potential diagnostic targets. Optimized parameters used in the analysis were: minimum length region of 200 bp, maximum entropy of 0.1, and segment gap limited to zero. Using Geneious Prime software, the conserved regions were also mapped to a panel of previously aligned sequences (n=11). The panel consisted of genomes of SARS-CoV-2 from Peru (MW030214, MW030206, MW030246, MW030232, and MW030200) and Wuhan (EPI_ISL_402124), and genomes of closely related viruses, comprising SARS-CoV (DQ898174), human coronavirus (NC_005831 and JN129835), MERS-CoV (KF192507), and bat coronavirus (FJ588686).

### RT-LAMP Primer Design

We designed RT-LAMP amplification primers for each conserved region using Primer Explorer v5 (https://primerexplorer.jp/e/). All the designed primer sets were first checked for primer dimerization using Multiple Primer Analyzer (ThermoFisher Scientific, Waltham, Massachusetts, USA). Interactions identified within and between the LAMP primer sets were analyzed using a relational table in Microsoft Excel.

Sets of RT-LAMP primers with the best parameters were selected. Each set included two outer primers (F3 and B3), two inner primers [forward inner primer (FIP) and backward inner primer (BIP)], and a loop forward (LF) and a loop backward (LB) primers, all of which were synthesized by Integrated DNA Technologies (IDT, Coralville, lowa, USA). The sequence and target region of each designed primer are shown in **Table S2**.

### One-Step Multiplex RT-LAMP Assay

The RT-LAMP assays were performed using lyophilized LAMP reagents from the Loopamp RNA/DNA amplification reagent D kit (Eiken Chemical Co. Ltd., Tokyo, Japan). First, each set of RT-LAMP primers was individually evaluated in simplex RT-LAMP assays in a 25 µL reaction mixture containing 1.6 µmol L^-1^ each of inner primers FIP and BIP, 0.2 µmol L^-1^ each of outer primers F3 and B3, 0.8 µmol L^-1^ each of loop primers LF and LB, and 5 µL of template RNA. The reagents were rehydrated with 18 µL of molecular grade water at room temperature. To determine the optimum temperature of each LAMP primer set, the reaction mixtures were incubated in a Loopamp real-time turbidimeter (LA-500; Eiken Chemical Co. Ltd., Tokyo, Japan) at 60°C, 63°C, and 65°C for 60 min and then at 80°C for 5 min to complete the reaction.

Second, we set up one-step multiplex RT-LAMP assays simultaneously targeting multiplex targets selected using the same conditions described above except that 8 µL of template RNA was used in the reaction. SARS-CoV-2 positive control (SPC) and non-template control (NTC) were included in each assay to confirm the performance of the reagents and the absence of contamination, respectively. To select and optimize the optimal primer set and reaction system, LAMP was assessed in real time by determining the amplification time (in minutes) and turbidity at 650 nm using the LA-500 turbidimeter. A reaction was considered positive when the turbidity reached 0.05 within 60 min, and by the color change from brown to green and by the laddering pattern of bands after gel electrophoresis.

### RT-qPCR Assay

The RT-qPCR assays were performed according to methods described by Corman et al. (2020) with slight modifications. In brief, RT-qPCR targeting the SARS-CoV-2-specific RNA-dependent RNA polymerase (*RdRp*) gene and human *GAPDH* (internal control) was performed in a 20 μl reaction mixture containing 5 μl of template RNA and the primers/probes in 1× CAPITAL qPCR Probe Mix (Biotechrabbit GmbH, Hennigsdorf, Germany). The probe targeting *RdRp* was fluorescence-labelled with FAM whereas the probe targeting *GAPDH* was fluorescence-labelled with ROX. Primer and probe sequences, as well as optimized concentrations, are shown in **Table S3**. RNA amplification was performed in a Rotor-Gene Q (Qiagen, Germantown, Maryland, USA) using the following cycling parameters: 50°C for 10 min and 95°C for 3 min, followed by 45 cycles at 95°C for 10 s and 58°C for 30 s.

### Analytical Sensitivity of the Multiplex RT-LAMP Assay

The sensitivity of the optimized RT-LAMP assay for detecting SARS-CoV-2 was determined using RNA obtained from Vero cell cultures of SARS-CoV-2, with initial titration from 6.5×10^6^ PFU/mL, that was provided by Laboratorio de Metaxénicas Virales of NIH-Peru. The viral RNA was 10-fold serially diluted up to 10^−8^ and processed for parallel testing involving multiplex RT-LAMP and RT-qPCR assays. The LoD was determined by identifying the lowest concentration of SARS-CoV-2 RNA at which ≥95% of 20 replicates showed positive results.

To determine the sensitivity of the multiplex RT-LAMP assay in conditions simulating the laboratory-based detection process, we used RNA samples obtained from nasal and pharyngeal swabs that were positive for SARS-CoV-2 RNA according to RT-qPCR, with high viral load (CT=12.48). The RNA samples were 10-fold serially diluted up to 10^−7^. The samples were then tested in parallel by multiplex RT-LAMP and RT-qPCR assays. The LoD of the multiplex RT-LAMP assay was then determined, and this was compared with that obtained by RT-qPCR.

Five microliters of each RT-LAMP reaction were electrophoresed on a 3% agarose gel in 1× TBE buffer (89mM Tris, 89mM boric acid, 2mM EDTA) at 110 V for 70 min. Reactions were considered positive for RT-LAMP products if they had both a color change from brown to green and a laddered banding pattern on agarose gel after electrophoresis.

### Analytical Specificity of the Multiplex RT-LAMP Assay

Cross-specificity tests for the multiplex RT-LAMP assay were carried out using viral and bacterial pathogens associated with respiratory infections, comprising influenza A (H1N1/H3N1/H3N2) virus (FluA), influenza B (Yamagata/Victoria) virus (FluB), respiratory syncytial virus (RSV), *Bordetella pertussis* (Bp), *Haemophilus influenzae* (Hi), *Neisseria meningitidis* (Nm), and *Streptococcus pneumoniae* (Spn). In addition, human immunodeficiency virus (HIV) and Zika virus (ZV) were included. RNA or DNA had previously been quantified using a Qubit 4 Fluorometer (ThermoFisher Scientific) and concentrations were adjusted to 1–2 ng/µL.

To evaluate the analytical *in silico* specificity, the final primer sets were analyzed with BLASTn using a high-priority pathogens database that included other closely related coronaviruses (**Table 1**). To do this, we downloaded pathogen genomes from the National Center for Biotechnology Information (NCBI) database and created a local database. Search parameters were automatically adjusted for short input sequences, query coverage was >60%, and the e-value was 10^-03^.

**Table 1:** High-priority pathogens and coronaviruses closely related to SARS-CoV-2 used in the alignment with the final LAMP primer sets.

High-priority organism	GenBank ID
Human coronavirus 229E	AF304460
Human coronavirus OC43	MN026164
Human coronavirus HKU1	MH940245
Human coronavirus NL63	NC_005831
SARS coronavirus GD01	AY278489
MERS coronavirus	KJ477102
SARS coronavirus ZMY 1	AY351680
Middle East respiratory syndrome-related	MG987420
Adenovirus	MF3585566
Human metapneumovirus (hMPV)	KY474545
Parainfluenza virus 1	JQ901998
Influenza A	MN423725
Enterovirus (EV68)	KT835407
Respiratory syncytial virus	RSU39661
Rhinovirus	NC_001617
*Chlamydia pneumoniae*	HV214386
*Haemophilus influenzae*	CP009610
*Legionella pneumophila*	CP016029
*Mycobacterium tuberculosis*	NC_018143
*Streptococcus pneumoniae*	NZ_CP007593
*Streptococcus pyogenes*	NZ_CP010449
*Streptococcus salivarius*	NZ_CP04084
*Bordetella pertussis*	NZ_CP038790
*Mycoplasma pneumoniae*	NZ_CP014267
*Pneumocystis jirovecii*	MH010446
*Candida albicans*	PRJNA231221
*Pseudomonas aeruginosa*	CP059852
*Staphylococcus epidermis*	CP061029

### Evaluation of Repeatability, Reproducibility, and Robustness

Precision was assessed using log-dilutions (10^-1^–10^-5^) of RNA extracted from cell culture of SARS-CoV-2 with initial titration from 6.5×10^6^ PFU/mL. Assays were performed by testing 3 and 6 replicates within and between runs, respectively, and by different operators, to calculate intra-assay precision (repeatability) and inter-assay precision (reproducibility), respectively, by means of the coefficient of variation (CV).

The robustness of the method was assessed by varying the concentrations of primers (0.75×, 0.5×, and 0.4× of the optimal concentration) and testing at two LAMP amplification temperatures (63°C and 65°C). Assays were performed using a 10^-1^ RNA log-dilution in duplicate under identical conditions (regarding the operator, equipment, and laboratory).

### Diagnostic Validation of the Multiplex RT-LAMP Assay

To evaluate the multiplex RT-LAMP assay using the final combined primer sets, we used 278 RNA samples obtained from nasal and pharyngeal swabs collected from individuals who tested positive (n=139) and negative (n=139) for SARS-CoV-2 RNA based on RT-qPCR (gold standard). These samples were randomly selected on different days and tested in parallel by both RT-qPCR and RT-LAMP assays.

The multiplex RT-LAMP assays were carried out using 8 μL RNA of each sample per reaction in a simple thermoblock (ThermoMixer C, Eppendorf Ltd., Hamburgo, Germany), as described above. The double-blind clinical evaluation was performed on COVID-19 predefined status and on reference test results. The RT-LAMP results were visually judged based on a color change from brown to green by two individuals, only being validated when the assessments of both individuals were the same. Sensitivity, specificity, positive predictive value (PPV), and negative predictive value (NPV) were determined to assess the diagnostic accuracy of the multiplex RT-LAMP compared to RT-qPCR (gold standard) according to the methodology described by Trevethan (2017).

### Statistical Analysis

Statistical analysis was performed using Stata/MP v15.0. One-way analysis of variance (ANOVA) test was used to assess for statistical significance of the differences between the different amplification times of the LAMP primers sets. Precision was determined by obtaining mean time-to-detection values and standard deviations (SD) of each set of replicates at a given concentration and calculating coefficients of variation (CV = SD/mean) (de Paz et al., 2020). Positive and negative results according to the RT-LAMP and RT-qPCR assays were analyzed using a 2×2 contingency table. Agreement analysis was performed using kappa concordance coefficients (Cohen’s kappa; ≥0.75 was considered good) and percentage agreement (≥0.9 was considered good) (Hu et al., 2020). We calculated 95% confidence intervals (CI), and 2-sided p<0.05 was considered significant for all statistical analyses.

## Results

### Screening of New WGS-Based Diagnostic Targets

**Figure 1:**
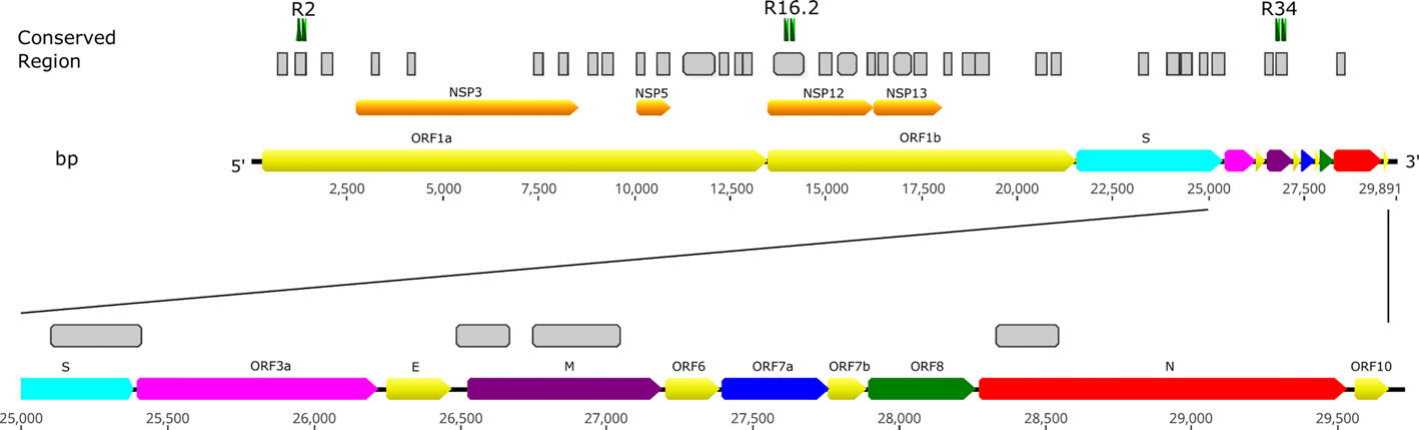
Locations of conserved regions that are potential diagnostic targets, identified by SARS-CoV-2 genome-wide comparison. LAMP primer sets designed for regions R2, R16.2, and R34 were combined in the multiplex RT-LAMP assay and resulted in sensitive and specific detection of SARS-CoV-2. Bp, base pair.

**Figure 2:**
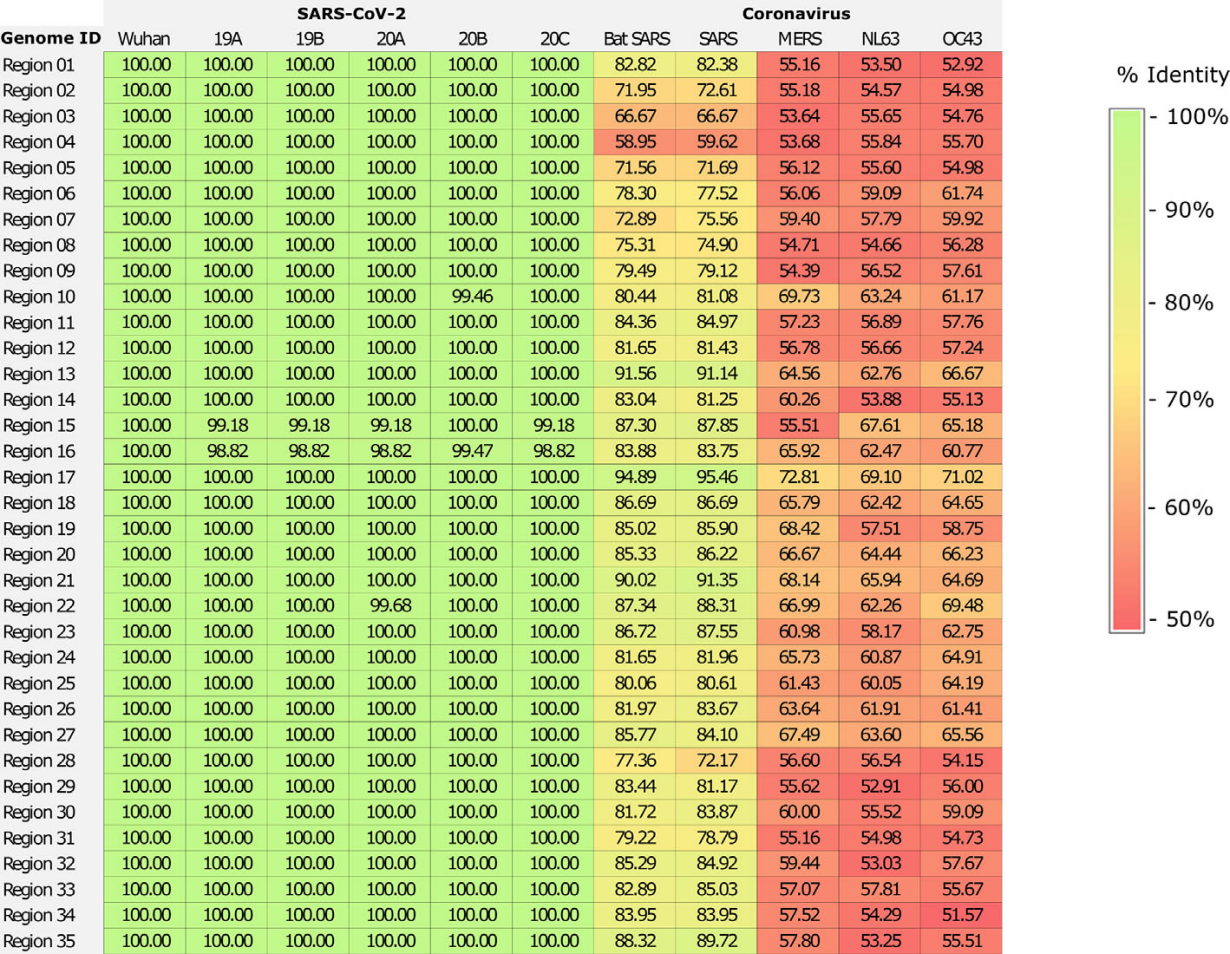
Similarity matrix of conserved region alignments involving SARS-CoV-2 genomes and other coronavirus sequences. The alignment included SARS-CoV-2 genomes from Wuhan (EPI_ISL_402124) and of five genetic clades circulating in Peru (19A [MW030214], 19B [MW030206], 20A [MW030246], 20B [MW030232] and 20C [MW030200]), and genomes of closely related viruses, comprising SARS-CoV (DQ898174), human coronavirus (NC_005831 and JN129835), MERS-CoV (KF192507), and bat coronavirus (FJ588686).

We sequenced 48 SARS-CoV-2 isolates circulating in Peru, yielding genomes with high coverage (mean depth = 2,767.68; mean reads per sample = 887,561). To select new diagnostic targets for identifying SARS-CoV-2 infections, we also analyzed 23,413 SARS-CoV-2 isolates circulating in different areas of the world, together with the isolates from Peru, obtaining a total of 35 highly conserved regions. The conserved regions were widely distributed throughout the SARS-CoV-2 genome. They are located in both independent and specific genes that encode either structural or non-structural proteins, including open reading frame 1ab (*ORF1ab*) and the spike (*S*), membrane (*M*), and nucleocapsid (*N*) genes, while only one sequence was identified among the *Spike-ORF3a* genes (**Figure 1** and **Table S1**). Most of the conserved regions had high similarity to SARS-CoV-2 genomes (100% identity) and, on average, 68% nucleotide identity with other coronavirus sequences (**Figure 2**).

### RT-LAMP Primer Design and Evaluation

**Figure 3:**
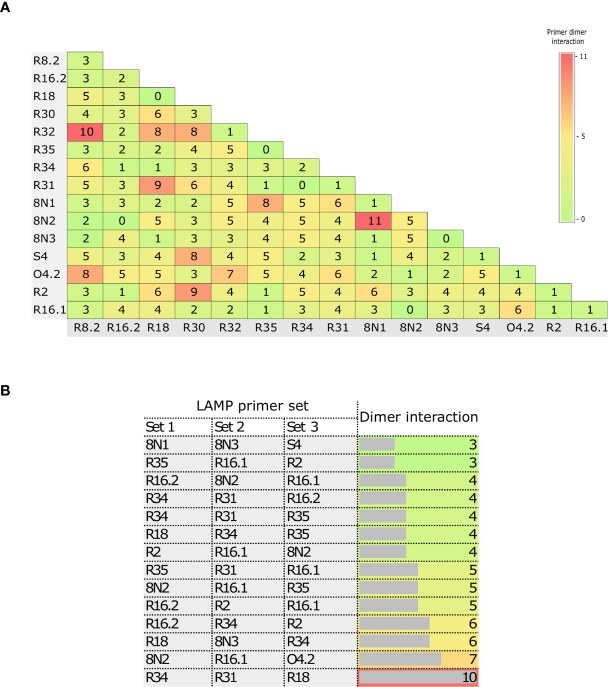
Matrix of the numbers of primer dimer interactions within and between LAMP primer sets. Dimer interactions within primer sets and between two primer sets **(A)**, and between three primer sets **(B)**.

The high number of LAMP primers increases the likelihood of the formation of primer dimers, which can impact assay sensitivity. Thus, we evaluated the interactions within and between the LAMP primer sets (**Figure 3**). There were few or no dimer interactions within primer sets (0–5), demonstrating that the primer design was optimal (**Figure 3A**). In addition, the number of interactions largely ranged from 0 to 11 (**Figure 3A**) between 2 LAMP primer sets, and from 3 to 10 (**Figure 3B**) among 3 LAMP primer sets.

**Figure 4:**
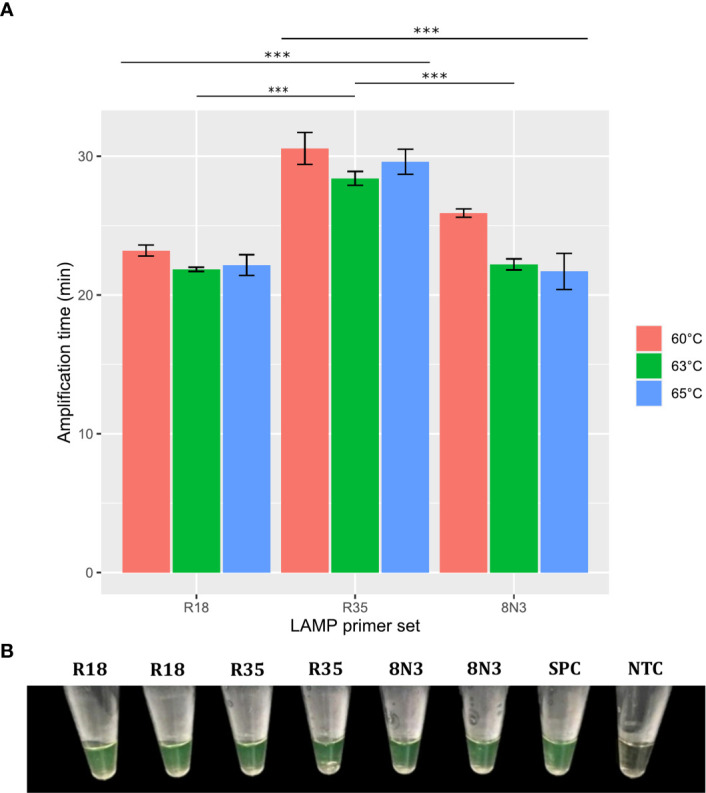
Effect of temperature and R18, R35, and 8N3 primer sets on RT-LAMP assay performance. Error bars represent the standard deviations of the amplification time mean from independent experiments performed in duplicate. ***P < 0.001. Significance analyzed by one-way analysis of variance (ANOVA) **(A)**. RT-LAMP assays using the R18, R35, and 8N3 primer sets for SARS-CoV-2 detection were considered positive when the solution color changed from brown to green **(B)**. NTC, negative non-template control; SPC, SARS-CoV-2 positive control.

**Figure 5:**
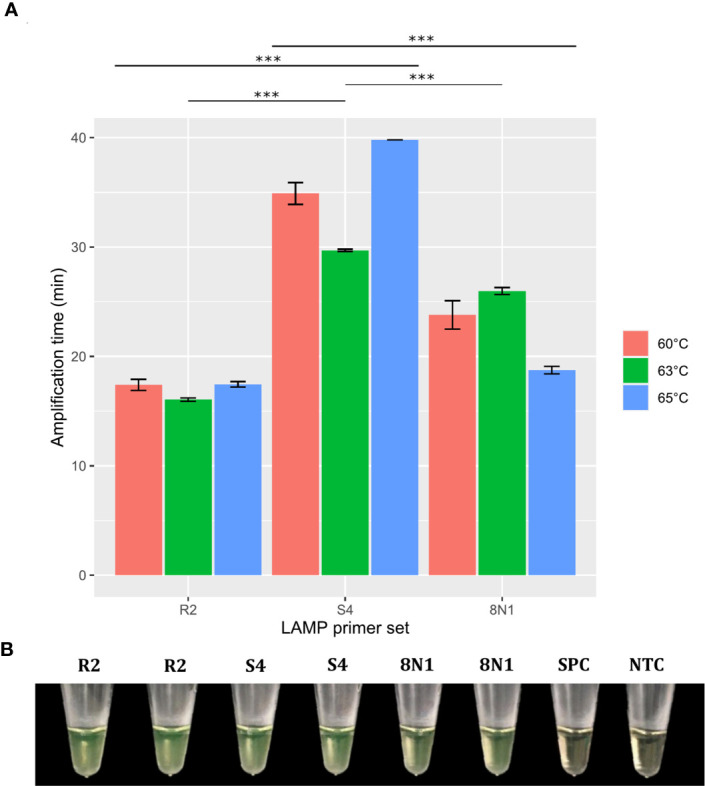
Effect of temperature and R2, S4, and 8N1 primer sets on RT-LAMP assay performance. Error bars represent the standard deviations of the amplification time mean from independent experiments performed in duplicate. ***P < 0.001. Significance analyzed by one-way analysis of variance (ANOVA) **(A)**. RT-LAMP assays using the R2, S4, and 8N1 primer sets for SARS-CoV-2 detection were considered positive when the solution color changed from brown to green **(B)**. NTC, negative non-template control; SPC, SARS-CoV-2 positive control.

**Figure 6:**
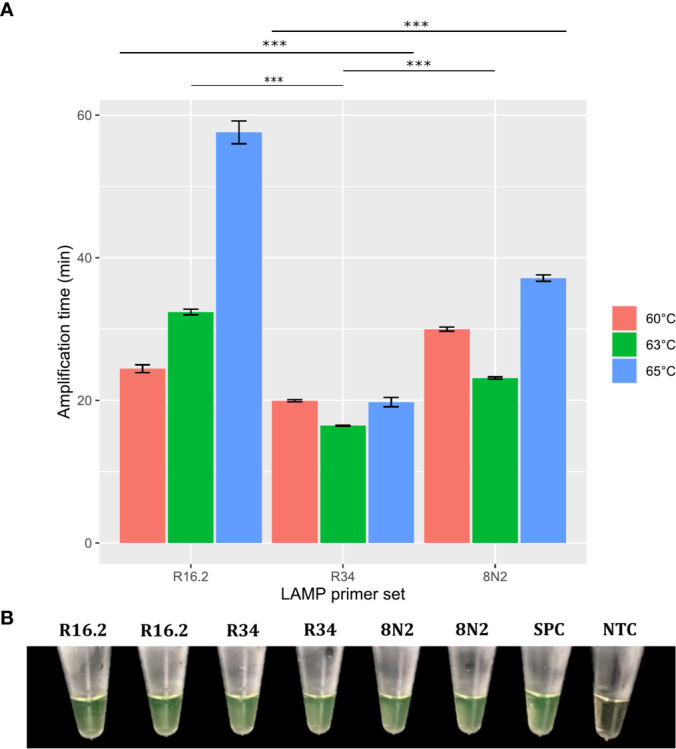
Effects of temperature and R16.2, R34, and 8N2 primer sets on RT-LAMP assay performance. Error bars represent the standard deviations of the amplification time mean from independent experiments performed in duplicate. ***P < 0.001. Significance analyzed by one-way analysis of variance (ANOVA) **(A)**. RT-LAMP assays using the R16.2, R34, and 8N2 primer sets for SARS-CoV-2 detection were considered positive when the solution color changed from brown to green **(B)**. NTC, negative non-template control; SPC, SARS-CoV-2 positive control.

**Figure 7:**
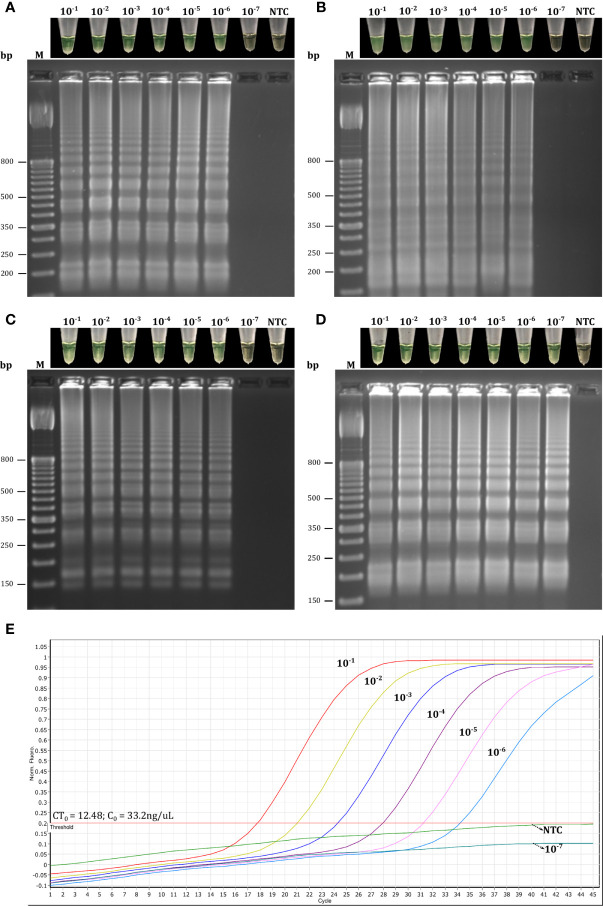
Evaluation of the single LAMP primer sets based on target dilution. Reactions using primer sets R18 **(A)**, 8N3 **(B)**, R2 **(C)**, and R34 **(D)** were considered positive for RT-LAMP products if they had both a color change from brown to green and a laddered banding pattern on agarose gel after electrophoresis. Target dilution was based on log10 dilutions (10^-1^ to 10^-7^) of SARS-CoV-2 RNA obtained from clinical samples and the samples were analyzed in parallel by simplex RT-LAMP **(A–D)** and RT-qPCR **(E)** assays. CT_0_, initial cycle threshold value of the clinical samples obtained by RT-qPCR; C_0_, initial concentration of the clinical samples; NTC, negative non-template control; bp, base pair.

**Figure 8:**
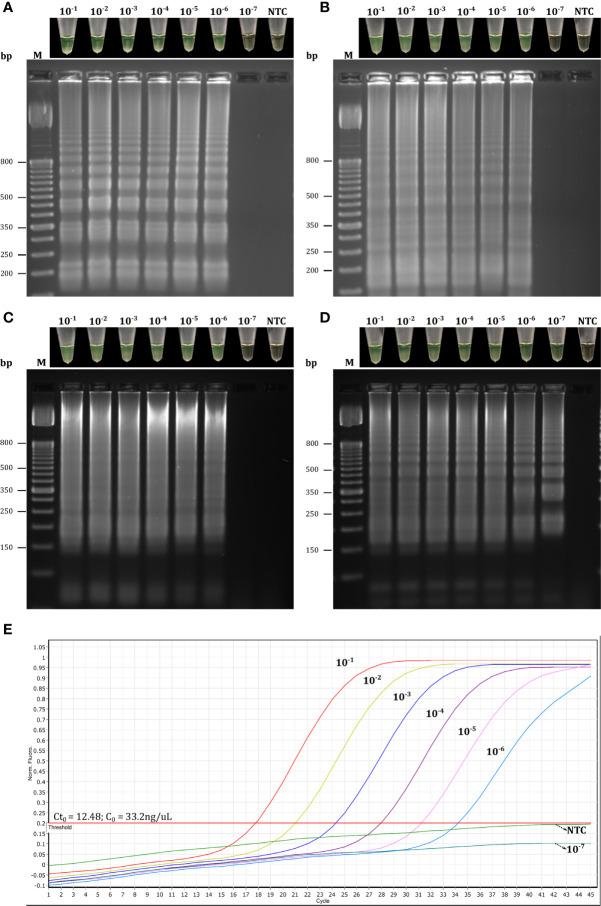
Evaluation of combined LAMP primer sets based on target dilution. Reactions using the combined primer sets R34-R31 **(A)**, R18-R34 **(B)**,R18-8N3-R34 **(C)**, and R16.2-R34-R2 **(D)** were considered positive for RT-LAMP products if they had both a color change from brown to green and a laddered banding pattern on agarose gel after electrophoresis. Target dilution was based on log10 dilutions (10^-1^ to 10^-7^) of SARS-CoV-2 RNA obtained from clinical samples and the samples were analyzed in parallel by multiplex RT-LAMP **(A–D)** and RT-qPCR **(E)** assays. CT_0_, initial cycle threshold value of the clinical samples obtained by RT-qPCR; C_0_, initial concentration of the clinical samples; NTC, negative non-template control; bp, base pair.

Based on these results, we first selected primer sets with 0–2 dimer interactions within each primer set, resulting in 12 LAMP primer sets being selected (**Figure 3A** and **Table S2**). These sets were then individually evaluated in RT-LAMP assays. Amplification yielding the best detection time (without amplification of the NTC) was achieved using the R18 (μ = 22.85 ± 0.29 min), 8N3 (μ = 22.2 ± 0.78 min), R2 (μ = 16.05 ± 0.29 min), and R34 (16.45 ± 0.10 min) primer sets at an optimal temperature of 63°C (**Figures 4**–**6**). All primer sets were able to detect SARS-CoV-2 RNA from clinical samples up to a log-dilution of 10^-6^ (CT=12.48), except the R34 primer set, which detected viral RNA diluted up to a log-dilution of 10^-7^. Conversely, RT-qPCR could only detect SARS-CoV-2 RNA up to a log-dilution of 10^-6^. This suggested that RT-LAMP using the R34 primer set is more sensitive than the reference test (**Figure 7**). Then, we selected the primer sets (n=10) that, when combined in groups of 2 and 3 led to only 0–1 or 0–10 interactions, respectively, and including the previously evaluated primer sets, to be used in multiplex RT-LAMP assays and ensure high performance (**Figure 3**). The combined primer sets that exhibited the best time for detecting SARS-CoV-2 RNA diluted up to 10^-6^ were R34-R31, R18-R34, and R18-8N3-R34, while R16.2-R34-R2 detected viral RNA diluted up to 10^-7^ at an optimal temperature of 63°C, in contrast with RT-qPCR, which only detected RNA diluted up to 10^-6^. Amplification of the NTC was not observed in any of these assays (**Figure 8**).

**Figure 9:**
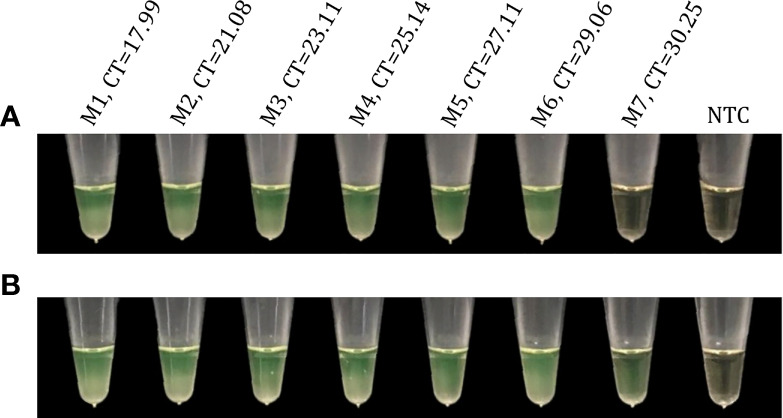
Evaluation of candidate primer sets for SARS-CoV-2 detection using a clinical sample panel with a viral load gradient. Reactions using the candidate primer sets R18-8N3-R34 **(A)** and R16.2-R34-R2 **(B)** were considered positive for RT-LAMP products if they had a color change from brown to green. Viral RNA obtained from clinical samples were analyzed in parallel by multiplex RT-LAMP and RT-qPCR assays. M1-M7, clinical samples with different cycle threshold (CT) values obtained by RT-qPCR; NTC, negative non-template control.

To select the final primer sets, the candidate sets were evaluated by multiplex RT-LAMP using a panel of SARS-CoV-2 RNA obtained from clinical samples (n=7) with a viral load gradient. Both the R18-8N3-R34 and R16.2-R34-R2 combined primer sets were able to detect viral RNA in a comparable time period. However, only the latter managed to amplify SARS-CoV-2 RNA in a sample with a low viral load (CT=30.25), coinciding with the RT-qPCR results (**Figure 9**). Finally, we selected the R16.2-R34-R2 combined primer sets to be used in the final multiplex RT-LAMP assay. The genes that they target span specific conserved sections of the SARS-CoV-2 genome. Specifically, the R16.2 and R34 sets target *RdRp* and *M*, respectively, while the R2 set targets *ORF1ab* (**Figure 1**).

### One-Step Multiplex RT-LAMP Analytical Performance

#### Analytical Sensitivity and Specificity

**Figure 10:**
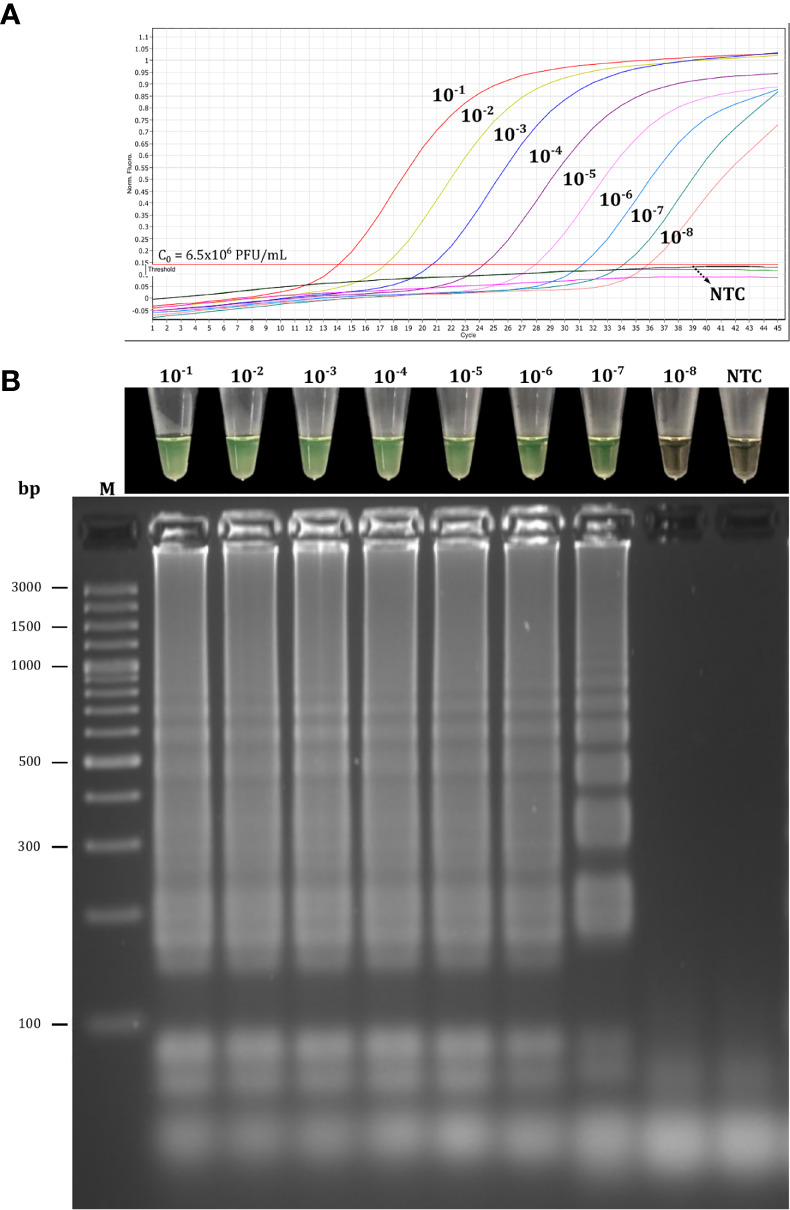
Analytical sensitivity of the multiplex RT-LAMP assay using SARS-CoV-2 RNA from cell culture. Limit of detection (LoD) was assessed using log-dilutions (10^-1^ to 10^-8^) of RNA extracted from SARS-CoV-2 from cell culture with initial titration (C_o_) from 6.5×10^6^ PFU/mL that were analyzed in parallel by the RT-qPCR **(A)** and RT-LAMP **(B)** assays. The LoD of the multiplex RT-LAMP assay was 0.65 PFU/mL (log-dilution 10^-7^). It was defined as the lowest concentration of SARS-CoV-2 RNA at which ≥ 95% of replicates (n = 20) showed positive results. RT-LAMP reactions were considered positive for SARS-CoV-2 RNA if they had both a color change from brown to green and a laddered banding pattern on agarose gel after electrophoresis. NTC, negative non-template control; M, molecular marker; bp, base pair.

The sensitivity of multiplex RT-LAMP was assessed using 10-fold serial dilutions of SARS-CoV-2 RNA obtained from Vero cell cultures of SARS-CoV-2. Based on colorimetric detection, the RT-LAMP assay was able to detect intact viral RNA concentration at log-dilutions of 10^−1^ to 10^−7^ (**Figure 10**). In addition, 20/20 replicates at log-dilution 10^-7^ were positive in <32 min, while 3/20 were positive at log-dilution 10^-8^ <35 min. Thus, the LoD for our multiplex RT-LAMP assay was 0.65 PFU/mL (log-dilution 10^-7^), which was the lowest concentration of SARS-CoV-2 RNA at which ≥95% of replicates showed positive results using the R16.2-R34-R2 combined primer sets. The lowest concentration to lead to a positive RT-qPCR result was 0.065 PFU/mL (log-dilution 10^-8^), indicating that the RT-qPCR assay is 10 times more sensitive than the multiplex RT-LAMP assay when RNA from cell culture of SARS-CoV-2 is used as a template. Nevertheless, when 10-fold serial RNA dilutions from clinical samples positive for SARS-CoV-2 RNA were used, the multiplex RT-LAMP assay could detect SARS-CoV-2 RNA diluted up to 10^-7^. This is in contrast to the RT-qPCR assays, which only detected RNA diluted up to 10^-6^, suggesting that RT-LAMP using the combined primer sets can amplify fewer copies of viral RNA from clinical samples, and in less time, than the reference test. This confirms that the assay has high clinical utility under real diagnostic conditions (**Figure 8**). In both cases, analysis by agarose gel electrophoresis revealed distinct banding patterns for the correct RT-LAMP reaction products (**Figures 8**, **10**).

**Figure 11:**
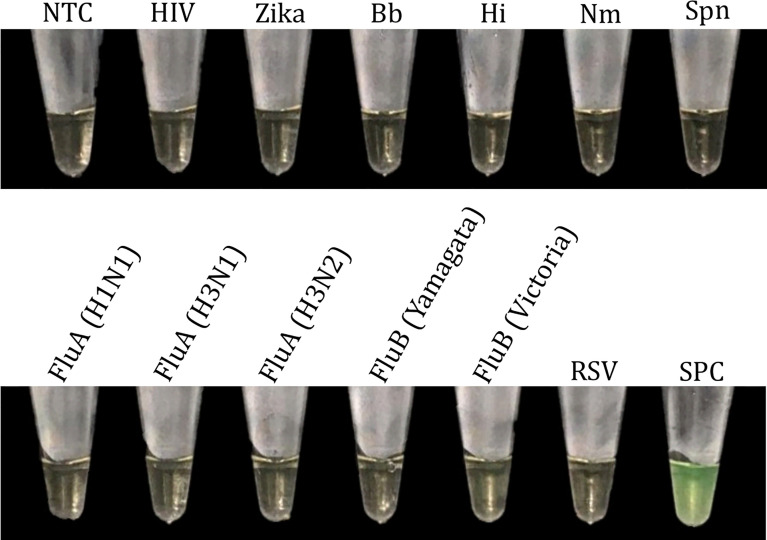
Analytical specificity of the multiplex RT-LAMP assay. HIV, human immunodeficiency virus; Zika, Zika virus; Bb, *Bordetella pertussis*; Hi, *Haemophilus influenzae*; Nm, *Neisseria meningitidis*; Spn, *Streptococcus pneumoniae*; Flu, influenza virus; RSV, respiratory syncytial virus; NTC, negative non-template control; SPC, SARS-CoV-2 positive control.

Furthermore, no cross-reactivity was found with either other viruses or bacteria, namely FluA (H1N1/H3N1/H3N2), FluB (Yamagata/Victoria), RSV, HIV, ZV, Bp, Hi, Nm, and Spn (**Figure 11**). Although the RNA sequences of other closely related coronaviruses were not included in this analysis, we performed an *in silico* cross-reactivity analysis by aligning the final primer sequences against the sequences of other closely related coronaviruses, as well as other unrelated viruses and bacteria likely circulating in Peru (**Table 1**). None of the analyzed primer sets matched any sequences included in the database, with the exception of a single primer (BIP-R16.2), for which 58% of nucleotides had 92% identity with sequences from other SARS coronaviruses. However, there is unlikely to be a risk of cross-reactivity, as LAMP amplicon generation is not possible with a single primer and multiple positive results based on a combination of each primer set are required to specifically identify SARS-CoV-2.

#### Repeatability, Reproducibility, and Robustness

Overall CV values of the multiplex RT-LAMP assay detection time regarding intra- and inter-assay precision were 4.75% and 8.30%, respectively (**Tables 2**, **3**). Intra-assay precision for the five SARS-CoV-2 RNA log-dilutions ranged from 3.78% at 10^-2^ to 7.59% at 10^-4^. The assay proved to have relatively high precision between runs at high, medium, and low concentrations, with inter-assay precision ranging from 7.27% at 10^-2^ to 12.74% at 10^-4^ (showing higher values at low concentrations).

**Table 2:** Intra-assay precision of the multiplex RT-LAMP assay.

Log-dilution SARS-CoV-2 RNA	No. of replicates	Mean Td (min)	SD	CV (%)
10^-1^	3	17.47	0.78	4.45
10^-2^	3	18.57	0.70	3.78
10^-3^	3	18.70	0.75	4.04
10^-4^	3	20.47	1.55	7.59
10^-5^	3	22.17	0.86	3.89
			Mean CV (%)	4.75

Td, time of detection; SD, standard deviation; CV, coefficient of variation.

**Figure 12:**
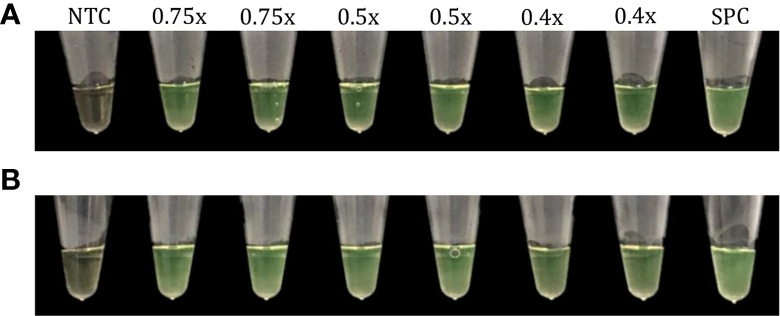
Evaluation of the robustness of the multiplex RT-LAMP assay. Variations in primer concentrations (0.75×, 0.5× and 0.4× of the optimal concentration) at 63°C **(A)** and 65°C **(B)**. Positive RT-LAMP reactions were visually determined based on a color change from brown to green. NTC, negative non-template control; SPC, SARS-CoV-2 positive control.

The multiplex RT-LAMP assays explored in this study were highly robust to changes in the primer concentrations, obtaining positive results in mean times of 18.65 min (0.75×), 20.90 min (0.5×), and 20.10 min (0.4×) at 63°C, and 20.90 min (0.75×), 22.35 min (0.5×), and 24.20 min (0.4×) at 65°C (**Figure 12**).

### One-Step Multiplex RT-LAMP Diagnostic Performance

To determine the specificity and sensitivity of the multiplex RT-LAMP assay, we tested 278 RNA samples obtained from nasal and pharyngeal swabs collected from individuals who tested positive (n=139) and negative (n=139) for SARS-CoV-2 RNA according to RT-qPCR (gold standard) at NHI-Peru. After parallel multiplex RT-LAMP and rt-RT-PCR assays, 139/278 samples were confirmed to be positive by RT-qPCR with cycle threshold (CT) values ranging from 21.97 to 43.43 (**Figure S1**). All these samples (n=139) were determined to be positive by the RT-LAMP assay results that were visually judged (based on a brown-to-green color change) following incubation for 45 min at 63°C. This confirmed the sensitivity of the multiplex RT-LAMP assay for detecting SARS-CoV-2 RNA in samples with a wide range of viral loads. We only found two discrepant results between the two assays, involving two RT-qPCR-negative samples that were determined to be positive in the multiplex RT-LAMP assay. To resolve these discrepancies, the two samples were retested by RT-LAMP and the reference test, confirming SARS-CoV-2 RNA detection in both samples based on RT-LAMP but no amplification by RT-qPCR. The overall sensitivity of the RT-LAMP assay was 100.0% (95% CI: 97.4–100.0%), and the specificity was 98.6% (95% CI: 94.9–99.8%) with a high degree of agreement between the two assays (Cohen’s kappa, 0.986; 95% CI: 0.966–1.000; p < 0.0001). PPV and NPV were 98.6% (95% CI: 95.0–99.8%) and 100.0% (95% CI: 97.3–100.0%), respectively.

## Discussion

In this study, we developed and evaluated the suitability of a multiplex RT-LAMP assay in a lyophilized format for detecting SARS-CoV-2 infection. We identified highly conserved regions among globally and locally detected SARS-CoV-2 isolates and we designed RT-LAMP primers targeting nonstructural protein 3 (NSP3), 5 (NSP5) and 13 (NSP13), RNA-dependent RNA polymerase (*RdRp*), spike (*S*), membrane (*M*), and nucleocapsid (*N*) genes (**Figure 1** and **Table S1**). Designs of primers and probes based on most of these targets have been recommended by the WHO for use in SARS-CoV-2 molecular diagnosis using RT-qPCR (Corman et al., 2020; Wang R. et al., 2020). However, primers used either in RT-qPCR or RT-LAMP assays have shown variable performance, mainly due to spurious formation of primer dimers, which may be a source of false positive results (Meagher et al., 2018; Park et al., 2020; Won et al., 2020). To overcome this limitation, we evaluated potential primer dimer formation within and between primer sets, selecting only the sets with 0–2 interactions within each set to be initially individually evaluated in RT-LAMP assays (**Figure 3A** and **Table S2**). Assays using primer sets R18 (*RdRp* gene), 8N3 (*ORF8-N*), R2 (*ORF1ab*), and R34 (*M* gene) efficiently amplified SARS-CoV-2 RNA (**Figures 4** **–** **6**) and were highly sensitive, with the best results obtained for the R34-based assay (**Figure 7**). These results are in agreement with previous research in which RT-LAMP assays targeting the *ORF1ab* gene took a mean ± SD time of 18 ± 1.32 min for detecting viral RNA among clinical samples, exhibiting higher sensitivity than conventional RT-qPCR (2×10^1^ *vs* 2×10^2^ copies/μL, respectively) (Yan et al., 2020). Conversely, a recent study demonstrated that RT-qPCR assays targeting the *RdRp* gene had the lowest sensitivity (probably due to a mismatch in the reverse primer with respect to the circulating SARS-CoV-2), and the study also indicated that *RdRp* was not a reliable target for detecting <1,000 viral RNA copies/μL of extracted nucleic acid (Vogels et al., 2020). Interestingly, PCR assays based on the *M* gene were more sensitive than those based on the *ORF1* and *RdRp* genes and proved to be suitable for detecting SARS-CoV-2 RNA in clinical samples with low viral loads (CT ~ 40) (Toptan et al., 2020). This is likely because the *M* RNA is one of the most abundantly expressed transcripts during viral replication (Kim et al., 2020). Thus, *M* might serve as a potential target for early detection of SARS-CoV-2. To improve the diagnostic performance, we set up a multiplex RT-LAMP assay based on combined primer sets, as previous studies have demonstrated that multiplexing led to higher sensitivity and specificity than individual gene detection (Elnifro et al., 2000; Yamazaki et al., 2010; Yamazaki et al., 2013). Among the 10 multiplex RT-LAMP assays, three had comparable analytical sensitivity to RT-qPCR (**Figure 8**), while the RT-LAMP assay involving the R16.2-R34-R2 combined primer sets had higher sensitivity to detect SARS-CoV-2 RNA in clinical samples (**Figures 8**, **9**). Therefore, these LAMP oligonucleotides were chosen as suitable diagnostic targets to be used in a one-step multiplex RT-LAMP assay. In contrast to previous studies that have used combined LAMP primer sets (Schermer et al., 2020; Thompson and Lei, 2020), the R16.2-R34-R2 combined primer sets target genes that are distributed throughout the SARS-CoV-2 genome, specifically targeting highly conserved regions of the SARS-CoV-2 *RdRp*, *M* and *ORF1ab* genes, respectively (**Figure 1** and **Table S2**). Although *RdRp* seems to be more polymorphic compared to other targets (Toptan et al., 2020), previous studies have demonstrated its high performance when it is used alone or in combination with other targets (Chan et al., 2020; Mohon et al., 2020). We found no differences in sensitivity when comparing single (R34) and multiplex (R16.2-R34-R2) RT-LAMP. However, we decided to use combined primer sets as it has been recommended to use at least two molecular targets to avoid potential cross-reaction with other endemic coronaviruses as well as potential genetic drift of SARS-CoV-2 (Tang et al., 2020).

The LoD of the multiplex RT-LAMP (0.65 PFU/mL, CT=34.12) was found to be slightly higher than the LoD of RT-qPCR (0.065 PFU/mL, CT=35.89) using SARS-CoV-2 RNA obtained from cell culture (**Figure 10**). Similar results were found in a study in which conventional RT-qPCR had a 10-fold lower detection rate compared to RT-LAMP results when using diluted viral RNA from cell culture of SARS-CoV-2. Interestingly, the CT values for the log-dilutions 10^-7^ and 10^-8^ were 34.2 and 37.8, respectively (Baek et al., 2020). These CT values are very close to the values obtained using our assays. Although there is debate about which CT values for a positive RT-qPCR result should be considered clinically relevant (Thi et al., 2020), there have been reports that CT values of 34.7–35.1 indicate very low RNA concentrations (10^2^ to 10^3^ copies/mL) (Ragan et al., 2020). Interestingly, most clinical samples that were positive for the other human SARS-CoV have been shown to have a viral concentration of 0.1 to 10^2^ PFU/mL, which encompasses the LoD determined in our multiplex RT-LAMP assay (Thai et al., 2004), suggesting that the methodology may be sufficient to confirm suspected cases at the early stage of SARS-CoV-2 infection. Conversely, when we tested dilutions of SARS-CoV-2 RNA obtained from clinical samples, the multiplex RT-LAMP assay was 10-fold more sensitive than the reference test (**Figure 8**). These results are in line with the LoD of 0.1 PFU obtained for a previous RT-LAMP assay developed for detecting SARS-CoV that demonstrated 100-fold greater sensitivity than conventional RT-qPCR (Thai et al., 2004), which is similar to the results of previous LAMP-based assays for diagnosis of SARS-CoV-2 infection (Hu et al., 2020; Yan et al., 2020). Differences in analytical sensitivity could have occurred because LAMP is more robust and more resistant to inhibitors and the genomic background of clinical samples in comparison to PCR (Notomi et al., 2000; Fakruddin et al., 2013).

The new multiplex RT-LAMP assay showed no cross-reactivity with other viruses and bacteria associated with respiratory diseases (**Figure 11**). In addition, the *in silico* specificity of the final primer sets was confirmed to be high, as the primers did not cross-react with other closely related coronaviruses or with pathogens likely circulating in Peru (**Table 1**), with the exception of BIP-R16.2, a small region of which matched with SARS coronavirus sequences. However, the use of the combined primer sets and the fact that LAMP amplification only occurs when six regions in a target DNA are recognized (Notomi et al., 2000) makes the new assay highly specific for SARS-CoV-2 detection in clinical samples.

Intra- and inter-assay precision of the multiplex RT-LAMP across the dilutions tested were shown to be suitable, obtaining mean CV values that did not exceed the recommended value of 15% (**Tables 2**, **3**) (Food and Drug Administration, 2018). Our CV values are lower than values of intra-assay precision (repeatability) reported for other RT-LAMP assays based on the *S* and *N* genes (16.96% and 15.91%, respectively) (Xing et al., 2020). This clearly demonstrates that our multiplex assay can provide acceptable sensitivity with relatively high precision for SARS-CoV-2 detection. Likewise, the assay showed a strong robustness across primer concentrations and temperatures (**Figure 12**), confirming the diagnostic advantage of LAMP compared to other nucleic acid sequence-based amplification methods (Notomi et al., 2000).

**Table 3:** Inter-assay precision of the multiplex RT-LAMP assay.

Log-dilution SARS-CoV-2 RNA	No. of replicates	Mean Td (min)	SD	CV (%)
10^-1^	6	16.12	1.62	10.03
10^-2^	6	17.48	1.27	7.27
10^-3^	6	17.40	1.57	9.02
10^-4^	6	18.60	2.37	12.74
10^-5^	6	20.32	2.18	10.73
			Mean CV (%)	8.30

Td, time of detection; SD, standard deviation; CV, coefficient of variation.

The multiplex RT-LAMP diagnostic sensitivity and specificity was 100.0% (95% CI: 97.4–100.0%) and 98.6% (95% CI: 94.9–99.8%), respectively, indicating higher performance than the performance of previously published RT-LAMP assays (Escalante-Maldonado et al., 2020; Hu et al., 2020; Thi et al., 2020). The high degree of agreement between the two assays (Cohen’s kappa, 0.986; 95% CI: 0.966–1.000; p < 0.0001) demonstrates that RT-LAMP is comparable to RT-qPCR for detecting SARS-CoV-2 in RNA samples isolated from nasal and pharyngeal swabs (Viera and Garrett, 2005). Furthermore, RT-qPCR itself does not reach a sensitivity of 100% (Ai et al., 2020), so cases can be missed by the reference test, in addition to some cases being identified as positive but being truly negative (although these false positives can be interpreted as a minor limitation). Reanalysis of the discordant results confirmed that the two RT-qPCR-negative samples remained positive in the multiplex RT-LAMP assay, suggesting that the multiplex methodology is more sensitive than RT-qPCR. A study of an RT-LAMP assay targeting the nonstructural protein 3 (NSP3) coding region of *ORF1ab* was positive for 10% (2/20) of RT-qPCR-negative samples, although one of the RT-qPCR-positive samples was negative based on RT-LAMP (Lamb et al., 2020). In our study, we did not obtain false negatives, which was likely due to simultaneous use of three targets, which increases the likelihood of detecting SARS-CoV-2 in samples with poor-quality RNA or a low viral copy number. Zhu et al. also demonstrated that an RT-LAMP assay had greater reliability and led to no false negatives when multiplex genes (*ORF1ab* and *N* genes) were targeted rather than a single gene (Zhu et al., 2020).

On the other hand, new molecular approaches based on CRISPR/Cas12 (Broughton et al., 2020) or Cas13a (Arizti-Sanz et al., 2020) have been developed for detecting SARS-CoV-2 in RNA extracted from nasopharyngeal or oropharyngeal swabs. Both assays demonstrate a high sensitivity and specificity. Nonetheless, they both have the drawback that the reaction tube has to be opened for lateral flow detection, increasing the likelihood of cross-contamination and false positives (Schermer et al., 2020). Recently a new molecular test based on LAMP and CRISPR/Cas12b have been developed for sensitive detection of SARS-CoV-2 in a single tube and by mean fluorescence readout. However, it requires additional reagents (e.g., fluorescent dyes) as well as a fluorescence reader which are sparse in resource-limited settings (Joung et al., 2020). Other methods to detect SARS-CoV-2 in nasal swabs are based on matrix-assisted laser desorption/ionization mass spectrometry (MALDI-MS) and machine learning analysis (Nachtigall et al., 2020) but the use of expensive equipment, special reagents, and complex algorithm-based detection make large-scale testing using these methods in developing countries less feasible.

Unlike other molecular approaches, our RT-LAMP assay comes with several clear-cut advantages. First, it only requires a simple thermoblock or water bath to maintain a constant temperature of 63°C for around 45 min, simultaneously detecting three SARS-CoV-2 targets, which provides early detection even at high CT values (>35) and could possibly mitigate the effects of SARS-CoV-2 genomic variants on molecular diagnostic. Second, the whole LAMP amplification reaction is carried out in a single tube and the results are based on rapid colorimetric detection without needing to use expensive fluorescence reagents and readers or open the amplification tube, facilitating simpler diagnostic testing with a low risk of cross-contamination. Third, the assay uses lyophilized LAMP reagents, which make it stable at room temperature and convenient for storage, transport, and operation in distant and resource-limited settings.

Although other types of clinical samples were not analyzed (e.g. sputum and saliva) in this study, nasopharyngeal swabs continue to be the best option for the detection of SARS-CoV-2 as they are easy to acquire and noninvasive, and they have been shown to have higher positive rate than sputum specimens in both RT-qPCR and RT-LAMP assays (Liu et al., 2020). Also, the sensitivity of RT-LAMP assays of saliva specimens varies, and these assays are reported to be inferior or not sufficiently reliable compared to RT-qPCR assays of saliva specimens for SARS-CoV-2 detection (Nagura-Ikeda et al., 2020; Thi et al., 2020).

On the basis of our data, we conclude that the new one-step multiplex RT-LAMP assay is amenable for identifying SARS-CoV-2-infected individuals with high and low viral loads (which are likely during early and later stages of the disease, respectively) using RNA isolated from nasal and pharyngeal swabs. Our results also indicate that the assay has highly similar diagnostic performance to conventional RT-qPCR, but it is faster, simpler, and cheaper. This makes the novel RT-LAMP assay very useful for reliable and timely diagnosis as well as for the epidemiologic surveillance of SARS-CoV-2 infection in primary health care units helping to control outbreaks during the pandemic. Finally, we argue that the multiplex RT-LAMP assay has the potential to be fully scaled up to simultaneously analyze large numbers of samples, especially if it is integrated into a point-of-care diagnostic device.

## Data Availability Statement

Study information is included in the article and **Supplementary Material**. Further inquiries can be made to the corresponding author. Genome sequences generated for this study can be found in NCBI GenBank (https://www.ncbi.nlm.nih.gov/sars-cov-2/) under accession no. MW030193–MW030240.

## Ethics Statement

This study was reviewed and approved by the Ethics Committee of the National Institute of Health of Peru (OI-011-20). Written informed consent for participation was not required for this study in accordance with the national legislation and the institutional requirements.

## Author Contributions

The study was planned and wrote by EJ-L. The collection of clinical samples was done by FV, NR, and MH. The identification of new targets and LAMP primer design were done by EJ-L and DT. Whole-genome sequencing and LAMP optimization experiments were done by EJ-L, LL and HH. EJ-L drafted the manuscript. All authors contributed to the article and approved the submitted version.

## Funding

This work was supported by the CONCYTEC-FONDECYT Program of Proyectos Especiales: Respuesta al COVID-19 2020-01-01 [grant number 034-2020-FONDECYT] and the National Institute of Health of Peru.

## Conflict of Interest

The authors declare that the research was conducted in the absence of any commercial or financial relationships that could be construed as a potential conflict of interest.
