# Supplementary material for: A Multiplex and Colorimetric Reverse Transcription Loop-Mediated Isothermal Amplification Assay for Sensitive and Rapid Detection of Novel SARS-CoV-2
Source: Front Cell Infect Microbiol. 2021 Jun 29;11:653616. doi: 10.3389/fcimb.2021.653616 (PMC8276080; doi:10.3389/fcimb.2021.653616)
Supplement: Supplementary file 1 [file DataSheet_1.pdf]

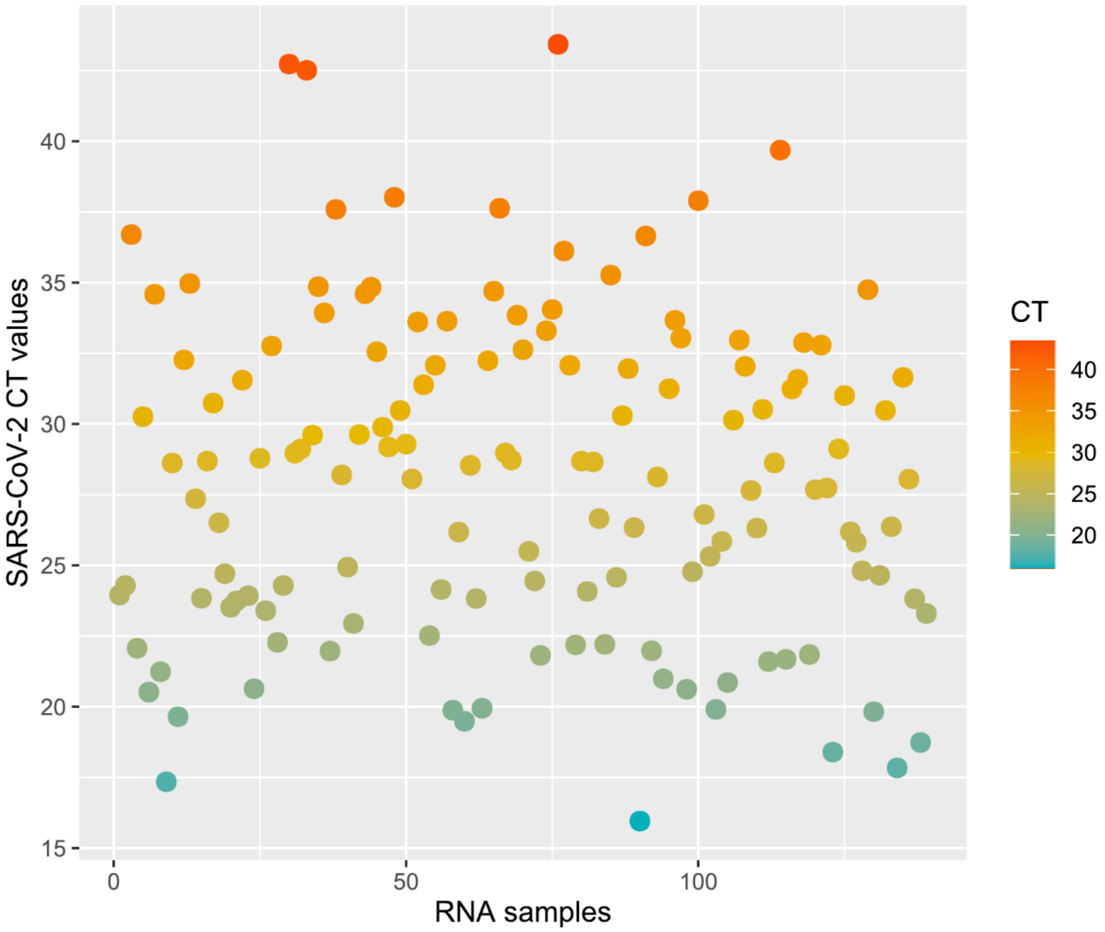

Figure S1. Distribution of the cycle threshold (CT) values of the RNA samples (n=139) which were positive for SARS-CoV-2 RNA by the RT-qPCR and multiplex RT-LAMP assays. RNA samples were isolated from nasal and pharyngeal specimens from patients with suspected COVID-19 and tested in parallel by both assays. The LAMP amplification reaction results were judged by simple visual assessment.

Table S1. Highly conserved regions selected as potential candidates for diagnosing SARS-CoV-2 infections.

Conserved region	Target	Position	
		Start	End
Region 1	ORF1ab	695	921
Region 2	ORF1ab	1137	1439
Region 3	ORF1ab	1815	2129
Region 4	ORF1ab	3145	3332
Region 5	ORF1ab	4074	4290
Region 6	ORF1ab	7357	7613
Region 7	ORF1ab	8032	8255
Region 8	ORF1ab	8783	9021
Region 9	ORF1ab	9161	9433
Region 10	ORF1ab	10071	10254
Region 11	ORF1ab	10607	10932
Region 12	ORF1ab	11292	12114
Region 13	ORF1ab	12215	12451
Region 14	ORF1ab	12623	12846
Region 15	ORF1ab	12848	13091
Region 16	ORF1ab	13651	14407
Region 17	ORF1ab	14806	15157
Region 18	ORF1ab	15325	15805
Region 19	ORF1ab	16082	16308
Region 20	ORF1ab	16392	16616
Region 21	ORF1ab	16796	17246
Region 22	ORF1ab	17306	17613
Region 23	ORF1ab	18065	18305
Region 24	ORF1ab	18563	18876
Region 25	ORF1ab	18915	19275
Region 26	ORF1ab	20488	20781
Region 27	ORF1ab	20909	21147
Region 28	Spike gene	23192	23402
Region 29	Spike gene	23905	24211
Region 30	Spike gene	24292	24570
Region 31	Spike gene	24750	24980
Region 32	Spike-ORF3a	25102	25406
Region 33	M gene	26488	26669
Region 34	M gene	26747	27045
Region 35	N gene	28332	28545

Table S2. Sequences in each LAMP primer set designed for detecting SARS-CoV-2.

**Primer**	**Region**	**Target**	**Sequence (5'–3')**	**bp**
F3-R16.2	Region 16	RdRp_ORF1ab	GAAAACCCAGATATATTACGCG	22
B3-R16.2			CTGGTCAAGGTTAATATAGGC	21
FIP-R16.2			CATGGCATCACAGAATTGTACTGTCGCCAACTTAGGTGAACG	42
BIP-R16.2			TGGTAACTGGTATGATTTCGGTGATCTACAACAGGAACTCCAC	43
LF-R16.2			TTTTAACAAAGCTTGGCGT	19
LB-R16.2			ATTTCATACAAACCACGCC	19
F3-R18	Region 18	RdRp_ORF1ab	TGTTCTTGCTCGCAAACA	18
B3-R18			TAACATTGGCCGTGACAG	18
FIP-R18			TCACTCAATACTTGAGCACACTCATCAACGTGTTGTAGCTTGTC	44
BIP-R18			ATGGTCATGTGTGGCGGTTCCTATTAGCATAAGCAGTTGTGG	42
LF-R18			AGCTAATCTATAGAAACGGTGT	22
LB-R18			CAGGTGGAACCTCATCAGGAG	21
F3-R32	Region 32	Spike-ORF3a	TCAATGAGGTTGCCAAGAA	19
B3-R32			AGGATCCACAAGAACAACA	19
FIP-R32			GCCAAATGTACCATGGCCATTTTATAATGAATCTCTCATCGATCTCC	47
BIP-R32			GGTTTTATAGCTGGCTTGATTGCCGCCCTTGAGACAACTACAG	43
LF-R32			CTGCTCATACTTTCCAAGTTCT	22
LB-R32			GTAATGGTGACAATTATGCTTTGC	24
F3-R35	Region 35	N	TCAGATTCAACTGGCAGTA	19
B3-R35			CCAATTTGGTCATCTGGACT	20
FIP-R35			ATTGGGTAAACCTTGGGGCCACCAGAATGGAGAACGCA	38
BIP-R35			CGTCTTGGTTCACCGCTCTCTAATTGGAACGCCTTGTCC	39
LF-R35			GACGTTGTTTTGATCGCG	18
LB-R35			ACTCAACATGGCAAGGAAG	19

F3-R34	Region 34	M	GCTATCGCAATGGCTTGTCT	20
B3-R34			GGTGTCCAGCAATACGAAGA	20
FIP-R34			AACGCGTACGCGCAAACAGTTGTAGGCTTGATGTGGCTCA	40
BIP-R34			TTCTCAACGTGCCACTCCATGGGGATCACAGCTCCGATTACG	42
LF-R34			CTGAAAGAAGCAATGAAGTAGC	22
LB-R34			CACTATTCTGACCAGACCG	19
F3-R31	Region 31	Spike	TTATGTCCCTGCACAAGAA	19
B3-R31			ACTGTGTTGTTGACAATTCC	20
FIP-R31			CCTTCACGAGGAAAGTGTGCTTAAGAACTTCACAACTGCTCC	42
BIP-R31			CACACACTGGTTTGTAACACAAAGGCAGTTACCAGACACAAATGTG	46
LF-R31			TTCCATCATGACAAATGGC	19
LB-R31			GAACCACAAATCATTACTACAGAC	24
F3-8N1	Region 8N	ORF8-N	AGATCACATTGGCACCCG	18
B3-8N1			CCATTGCCAGCCATTCTAGC	20
FIP-8N1			TCTGCGTAGAAGCCTTTTGGCACAATGCTGCAATCGTGCTAC	42
BIP-8N1			GGCGGCAGTCAAGCCTCTTCTTACTGCTGCCTGGAGTTG	39
LF-8N1			TGTTGTTCCTTGAGGAAGTT	20
LB-8N1			CGTTCCTCATCACGTAGTCGC	21
F3-8N2	Region 8N	ORF8-N	GGCTTCTACGCAGAAGGGA	19
B3-8N2			TTGCTCTCAAGCTGGTTCAA	20
FIP-8N2			AGTTCGTTTACTGCTGCCTGGAGCAGTCAAGCCTCTTCTCG	41
BIP-8N2			TCTCCTGCTAGAATGGCTGGCATCTGTCAAGCAGCAGCAAAG	42
LF-8N2			GTTGCGACTACGTGATGAGG	20
LB-8N2			TGGCGGTGATGCTGCTCTTG	20
F3-8N3	Region 8N	ORF8-N	TGGACCCCAAAATCAGCG	18
B3-8N3			GCCTTGTCCTCGAGGGAAT	19
FIP-8N3			CCACTGCGTTCTCCATTCTGGTAAATGCACCCCGCATTACG	41

BIP-8N3			CGCGATCAAAACAACGTCGGCCCTTGCCATGTTGAGTGAGA	41
LF-8N3			ACTGCCAGTTGAATCTGAGGG	21
LB-8N3			TACTGCGTCTTGGTTCACCGC	21
F3-S4	Region S4	Spike	TTACCCCCTGCATACACT	18
B3-S4			CAGTGGAAGCAAAATAAACACC	22
FIP-S4			GGTAAGAACAAGTCCTGAGTTGAATAATTCTTTCACACGTGGTG	44
BIP-S4			TCCAATGTTACTTGGTTCCATGCTGGACAGGGTTATCAAACCTC	44
LF-S4			ACTGAGGATCTGAAAACTTTGTCAG	25
LB-S4			TACATGTCTCTGGGACCAATGGTAC	25
F3-R2	Region 2	ORF1ab	GTGCCTTTCAACTCTCATG	19
B3-R2			CAAGACTATGCTCAGGTCC	19
FIP-R2			GCCACAAAATTCGCAAGTGGATTGTGGTGAAACTTCATGG	40
BIP-R2			TTGACTAAAGAAGGTGCCACTACTATTGTGACATGCTGGACA	42
LF-R2			AACAAAATCGCCCGTCTG	18
LB-R2			GTTACTTACCCCAAAATGCTG	21
F3-R16.1	Region 16	RdRp_ORF1ab	GAAAACCCAGATATATTACGCG	22
B3-R16.1			CTGGTCAAGGTTAATATAGGCA	22
FIP-R16.1			CATGGCATCACAGAATTGTACTGTTACGCCAACTTAGGTGAAC	43
BIP-R16.1			TCAATGGTAACTGGTATGATTTCGGTCTACAACAGGAACTCCACTA	46
LF-R16.1			TTTAACAAAGCTTGGCGT	18
LB-R16.1			GATTTCATACAAACCACGCC	20

Table S3. Primers and probes used in real-time RT-PCR (gold standard) for detecting SARS-CoV-2.

Gene	Primer/probe	5' fluorophore	Sequence	3' quencher	Concentration (μM)
	RdRp_SARSr-F		GTGARATGGTCATGTGTGGCGG		0.4
RdRp	RdRp_SARSr-R		CARATGTTAAASACACTATTAGCATA		0.4
	RdRp_SARSr-P2	FAM	CAGGTGGAACCTCATCAGGAGATGC	BHQ1	0.2
	GAPDH_F		GTGAAGGTCGGAGTCAACGG		0.2
GAPDH	GAPDH_R		TCAATGAAGGGGTCATTGATG		0.2
	GAPDH_P	ROX	CGCCTGGTCAACAGGGTCGC	BHQ2	0.1
